# How Are Local People Driving and Affected by Forest Cover Change? Opportunities for Local Participation in REDD+ Measurement, Reporting and Verification

**DOI:** 10.1371/journal.pone.0145330

**Published:** 2016-11-02

**Authors:** Indah Waty Bong, Mary Elizabeth Felker, Ahmad Maryudi

**Affiliations:** 1 Center for International Forestry Research, Jl. CIFOR, Situ Gede, Bogor 16115, Indonesia; 2 Faculty of Forestry, Universitas Gadjah Mada, Bulaksumur, Yogyakarta, Indonesia 55281; Wageningen University, INDONESIA

## Abstract

Deforestation and forest degradation are complex and dynamic processes that vary from place to place. They are driven by multiple causes. Local communities are, to some extent, driving and also affected by some of these processes. Can their knowledge aid and add to place-specific assessment and monitoring of Deforestation and forest Degradation (DD) drivers? Our research was conducted in seven villages across three provinces of Indonesia (Papua, West Kalimantan and Central Java). Household surveys and focus group discussions were used to investigate how local community knowledge of DD drivers contributes to place-specific assessment and monitoring of DD drivers. We analyzed the link between drivers and local livelihoods to see how attempts to address deforestation and forest degradation might affect local communities and how this link might influence their participation in climate change mitigation measures such as Reducing Emissions from Deforestation and Forest Degradation (REDD+) and Measuring, Reporting and Verifying (MRV) activities. We found that local knowledge is fundamental to capturing the variety of drivers particularly in countries like Indonesia where forest and socio-economic conditions are diverse. Better understanding of drivers and their importance for local livelihoods will not only contribute to a more locally appropriate design of REDD+ and monitoring systems but will also foster local participation.

## Introduction

There is increasing interest in the potential of local participation in the Reducing Emissions from Deforestation and forest Degradation, sustainable management of forests, conservation and enhancing carbon stock (REDD+) scheme [1–5]. Nonetheless, many studies have focused mainly on local participation in measuring and monitoring (M) of carbon stock [6–11]. While most agree that participatory Measurement, Reporting and Verification (MRV) is possible and can offer many benefits, e.g. lower costs but with equally reliable results compared to those produced by external experts [1, 4, 12–15], few studies have investigated local participation beyond measuring and monitoring carbon.

Interventions that can effectively address Deforestation and forest Degradation (DD) need to be designed based on comprehensive data and analysis on forest cover changes and what drives these changes [5, 16]. Meanwhile, forest cover changes are occurring at the local level, thus the factors that directly drive these changes and the underlying social, economic and/or political forces working behind these drivers, to some extent, are known locally. This information on local level land use activities is essential for place-specific assessment and analysis, and strategy development to address and monitor forest cover changes.

Studies on the drivers of DD are often based on global, regional or national-scale analyses of remote sensing data, documents and scientific literature [16–20]. There are some place-specific analyses identifying drivers at a smaller scale using remote sensing [21–24]. However, remote sensing data are limited because the image interpretations are highly dependent on the technical skills and knowledge of those analyzing the maps as well as other technical problems such as persistent cloud cover and costly high resolution images [25, 26]. Most importantly, in this type of study, the identified drivers might not fully capture the variety of local drivers, particularly those that are related to forest degradation such as selective logging and artisanal mining [27, 28]. Locally collected information can fill these gaps, providing better data on varieties of drivers and explaining the dynamic processes that led to forest cover changes.

In terms of prioritizing drivers, most studies assess the importance of drivers based on the scale of forest cover change; the bigger the deforested area caused by a certain driver, the higher the priority to monitor and address the driver. However, at the local level, each driver varies in its relevance to local livelihoods. Analyzing the link between drivers and local livelihoods is essential to understand the possible impacts of the activities, aiming to reduce DD, on local livelihoods and if local communities are to be involved in REDD+ activities and monitoring.

Indonesia, as part of its REDD+ readiness phase, is currently developing strategies to address drivers of DD and design an MRV system that also includes monitoring drivers of deforestation and forest degradation [16, 29]. Indonesia is regarded as one of the REDD+ participant countries with good quantitative data on drivers available at the national-scale [16]. Yet, the REDD+ interventions the Indonesia Government have proposed are considered to have no explicit linkage to DD drivers [30]. This link is important if interventions are to be successful in reducing DD. Meanwhile, at the project level the MRV capacity and readiness is low in terms of, among others, understanding drivers, interventions planned for the proximate and underlying drivers, and long-term monitoring plans to evaluate the interventions [31].

Given the potential of local participation in assessing and monitoring drivers of DD in the REDD+ MRV context, we assessed local perceptions of drivers of DD and investigated the relationship between drivers and local livelihoods. We highlight how attempts to address DD might affect local livelihoods and thus influence community participation in REDD+ activities in general and MRV specifically. Finally, we discuss how incorporating locally collected information to better capture the variety of drivers will benefit REDD+. While we acknowledge that local people also contribute to forest recovery or regrowth, this paper focuses only on the drivers of deforestation and forest degradation at the local level.

## Materials and Methods

### Study Areas

**Fig 1:**
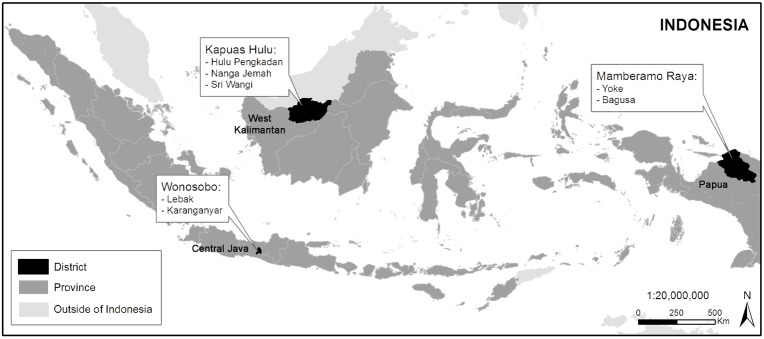
Map of the study sites. The map was developed by the Center for International Forestry Research under the Participatory Measurement, Reporting and Verification (CIFOR-PMRV) Research Project using administrative boundary data obtained from the Indonesian National Statistics Agency *(Badan Pusat Statistik Indonesia)* 2010.

We conducted our study in seven villages in three provinces of Indonesia: Yoke and Bagusa in Mamberamo Raya District, Papua; Hulu Pengkadan, Nanga Jemah and Sri Wangi in Kapuas Hulu District, West Kalimantan; and Lebak and Karanganyar in Wonosobo District, Central Java (Fig 1). In selecting the study sites, a set of criteria was used to represent various forest, social and economic conditions in Indonesia [32].

#### Mamberamo Raya, Papua

Yoke and Bagusa have a high percentage of natural forest cover, low population density and poor accessibility. The area has been of interest to many investors for its natural resources as shown by the past oil exploration activities, ongoing logging concessions and possibly natural gas and coal exploration in the future. Even though there is no formalized community forestry scheme present in the area, the villages have managed the forest, possibly for centuries, following their customary rules.

#### Kapuas Hulu, West Kalimantan

Hulu Pengkadan, Nanga Jemah and Sri Wangi have a medium percentage of natural forest cover, population density and accessibility. There were expansive logging activities in the past that have now been inactive for more than ten years, but the private concessionaire still holds an active logging license. Various companies have attempted to open oil palm plantations and map areas in the villages for mining exploration. Traditionally, forest areas have been managed by the villages and supported by customary institutions. Formalized community forestry schemes have recently been introduced with the designation of the Village Forest (*Hutan Desa*) areas in Nanga Jemah and Sri Wangi in 2014.

#### Wonosobo, Central Java

Lebak and Karanganyar are at the other extreme end of our study sites; the villages are densely populated, have high accessibility, but have almost no natural forest except for very small patches in the forest plantation areas, which have been designated for preservation. The forest plantation is managed by the state-owned forest company (Perum Perhutani) and consists of pine and other timber trees. Despite its low percentage of natural forest cover, the villages have a long history of community forestry as part of both the Perhutani’s community partnership program and agroforestry practices initiated by the community itself.

### Methods and Analysis

This study was part of a larger study that used a multidisciplinary approach (social, governance, remote sensing and geographical information systems) to look at the feasibility of Participatory Measurement, Reporting and Verification (PMRV) in Indonesia [32]. In this study, our aim was to better understand the DD drivers from the local communities’ perspective and what opportunities it provides for REDD+ PMRV.

The primary data were collected during two field visits in each village (7 villages in total) from July 2013 to January 2014 through Household Surveys (HHs) and Focus Group Discussions (FGDs).

#### Household livelihoods

Data on household livelihoods were derived from household surveys. We used the following formula to calculate the sample size for the household surveys in each village:

$$n=\frac{Np\left(1-p\right)x^{2}}{\left(N-1\right)\;\frac{c^{2}}{z^{2}}+p\left(1-p\right)}$$

n = minimum sample size

N = population size (number of households in a village)

z = normal score, determined by a confidence level of 95% = 1.96

c = margin of error (upper and lower bounds), determined by the level of accuracy we would like to have (0.1)

p = a rough estimate of the household proportion who were our main interest in the survey (0.7 and 0.8 for Bagusa and Yoke respectively and 0.5 for the other 5 villages. We used larger proportion values for Bagusa and Yoke because we considered the villages in Papua to be more homogenous in terms of people behavior and environmental conditions)

Since the villages consist of two or three hamlets the households were stratified by hamlets. The sampling stratification ensures proper representativeness of households from each hamlet. The households were then selected randomly using the lottery method of sampling [33]. We interviewed the first adult we met in each household. If no one was available at the time of our visit, we revisited the household. If we still found no eligible respondent or the household refused to participate, we randomly selected another household as a replacement. In total we conducted 418 household surveys (Papua: 34 HHs in Bagusa and 28 HHs in Yoke; West Kalimantan: 64 HHs in Hulu Pengkadan, 65 HHs in Nanga Jemah, and 56 HHs in Sriwangi; and Central Java: 78 HHs in Lebak and 84 HHs in Karanganyar). We used Microsoft Excel software [34] to code and run descriptive statistical analysis for the livelihoods data.

We defined a household livelihood as the income, both cash and subsistence, earned by a household through different means. For analysis, we classified the collected livelihood data into thirteen categories based on their relations to the forest from the most forest based livelihoods to non-forest related [35, 36]: 1) hunting and gathering, 2) shifting cultivation, 3) fishing, 4) harvesting timber from natural forest, 5) harvesting timber from forest plantations and agroforests, 6) harvesting non-timber forest products (NTFPs), 7) farming, 8) animal husbandry and fish farming, 9) artisanal gold mining, trade and small enterprise working with forest products, 10) working as labor in forest related activities, 11) working outside forest/agriculture/agroforestry sector, e.g. teachers, civil servants, and traders, 12) in-kind or cash aid, and 13) remittance.

In this study, ‘Agroforest’ (*agroforestri*, *kebun or talun*) is a term used in Java to describe a privately owned area planted with mixed timber and fruit trees as well as agricultural crops. ‘Garden’ (*kebun*) in West Kalimantan is a privately (individual, family or group) owned area with mixed planted and naturally growing fruit and timber trees, but commonly dominated by one species such as rubber, Borneo Tallow Nut (*tengkawang*) or durian. Grove *(dusun*) is a term used in Papua for a privately (family or clan groups) owned area that consists of planted and mixed fruit and timber trees and naturally occurring and/or planted sago trees. Non-Timber Forest Products (NTFPs) are broadly defined as all products, excluding timber, collected for both subsistence and cash income purposes that are derived from natural forests as well as agroforest, garden and grove.

#### Local perceptions of drivers of deforestation and forest degradation

We used FGD to assess the changes in village forests and factors that drive these changes. An FGD was conducted in each village (7 in total) with a mix of 5–15 women and men participating in each FGD. The participants were suggested by the villagers during a community meeting based on their knowledge of the forest condition and changes in the village. A research team of at least 3 people facilitated each FGD; a facilitator led the discussion with the help of a co-facilitator and another team member observed and documented the process.

We used ‘forest’ as commonly defined by the local communities. In Central Java, villagers use ‘forest’ when talking about Perhutani’s forest plantation(s), predominately covered in pine trees mixed with timber trees such as teak, mahogany, and albizia. In West Kalimantan and Papua, ‘forest’ means an area covered in a mix of natural growth (primary forest) or re-growth trees (secondary forest).

Drivers of DD were assessed using the ‘problem tree’ method in which participants were asked to identify and discuss causes of DD in their village. The causes were written down and visualized in the form of a tree-like diagram to represent cause-effect relationships that led to forest decline in their area. The facilitator then distributed five stickers to each participant; each sticker represented one point. Each participant was asked to select and score drivers that she/he thought had significant impact on forest decline by placing one or more stickers (up to five stickers) on each selected driver. The bigger its perceived contribution to forest decline, the more stickers a driver got. We used the terms ‘cause’ for ‘driver’ and ‘forest decline’ for ‘deforestation and forest degradation’ interchangeably.

We usedan R-based Qualitative Data Analysis (RQDA) software [37] to code and analyze the drivers and descriptive explanation given during the FGDs. The participants identified direct causes of forest decline and indirect factors behind the direct causes. In the analysis, we re-defined these two categories, i.e. direct causes of proximate drivers and the indirect causes of underlying factors. The content in these two categories and links between them are the same as those generated in the FGDs [19]. Proximate drivers are human activities conducted at the village level and have direct impact on forest cover. Underlying factors are social, economic, and/or political forces behind proximate drivers working either at the local level or as a result of multi-scale processes. Natural disasters (e.g. erosion, storm), natural processes (e.g. tree competition) and tree diseases were mentioned in some villages as causes of forest decline, but for analysis we only focused on those that were anthropogenic. Next, in the analysis, we ranked the proximate drivers from the highest to the lowest in terms of impact on forest decline based on the sum of the scores given to proximate drivers and their related underlying factors. When one underlying cause was mentioned for two or more proximate causes, the score was divided proportionally among the proximate drivers. Some proximate drivers are un-ranked because during the scoring activity, none of the participants gave these drivers a score (the participants did not consider these as having a significant impact on forest decline in comparison to other drivers).

#### Relative importance of drivers of deforestation and forest degradation for local livelihoods

To assess the relative importance of drivers for the local livelihoods, we followed three steps. First, we clustered proximate drivers and underlying factors together based on the respective livelihood activity they were related to. In the FGDs, the participants were asked to identify and explain the drivers. Information on livelihood activities that were related to drivers was collected during this exercise. Second, we estimated the percentages of households that depended on each of these livelihoods (drivers) and its mean rank in the household livelihoods in each village. Data on the percentages of households and rank were derived from the household surveys where respondents were asked to list and rank all sources of income (livelihood) in his/her household from the highest to the lowest based on its contribution to the whole household income. Finally, we mapped the position of each livelihood (driver) in a village within four quadrants of relative importance: 1) high importance, 2) medium importance-1, 3) medium importance-2, and 4) low importance. The quadrants are defined by two dimensions: one reflects the proportion of households dependent on a particular livelihood that is also identified as a driver in a village, and the other indicates the mean rank of that livelihood in the households’ livelihoods within a village. The first dimension is plotted along the horizontal axis as a percentage of households whose livelihoods are also drivers of DD, and the second dimension is plotted along the vertical axis as the mean rank of the drivers’ contribution to the households’ livelihoods in a village. We utilized Microsoft Excel software [34] to build and present the importance quadrant map.

### Ethics Statement

Prior to our research we obtained a written research permit from the Directorate General of National Unity and Politics (*Kesatuan Bangsa dan Politik*, *Kesbangpol)* of the Indonesia Ministry of Home Affairs. We reported to the provincial, district, and sub-district government and police offices where the studies were conducted. The village level governments then granted our team verbal permission to conduct our research. At the beginning of each fieldwork visit we held a village level community meeting to introduce the research team, explain the study objectives and activities as well as to ask the community for permission to conduct the research activities. In some villages where hamlets are some distance from each other, community meetings were held in each hamlet. During household surveys, we re-explained to the participants about the study, its objectives, household selection, survey processes, voice recordings, confidentiality, and how the data would be used. We then obtained verbal informed consent from respondents before we continued with the household surveys. We audio recorded the interviews except for respondents who requested not to be recorded. In our analysis, all data was rendered anonymous to protect the respondents’ confidentiality.

## Results

### Local livelihoods

**Fig 2:**
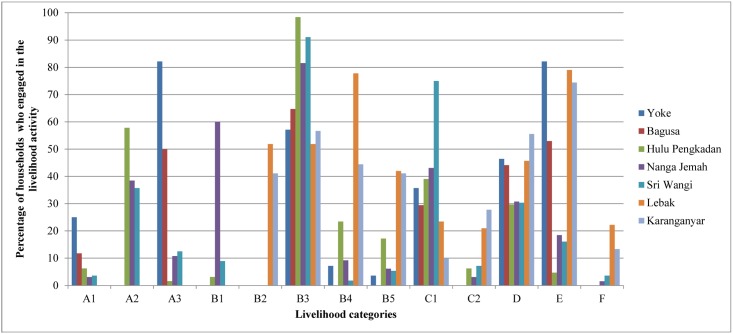
Proportion of households by livelihood categories in each village (n = 418 HHs). Livelihood categories: A1: Hunting and gathering, A2: shifting cultivation, A3: fishing, B1: harvesting timber from natural forest, B2: harvesting timber from plantation forest and agroforest, B3: harvesting NTFPs, B4: farming, B5: animal husbandry, fish farming, C1: artisanal gold mining, trade and small enterprise working with forest products, C2: labor in forest related activities, D: working outside forest/agricultural/agroforestry sectors, e.g. teacher, civil servant, trader, etc., E: in-kind or cash assistance, F: remittance.

In the villages, there were multiple sources of livelihoods ranging from highly forest dependent (e.g. hunting and gathering forest products) to non-forest related (e.g. teachers and remittance from relatives) (Fig 2).

#### Forest dependent livelihoods

Forest plays an important role in the household income for subsistence and cash across all the study villages. Fig 2 shows the diversity of forest-related livelihood activities and percentages of households relying on these livelihoods across seven villages.

Fishing and collecting Non-Timber Forest Products (NTFPs) were the dominant forest-based livelihoods in both villages in Papua. Approximately 50% of households in Yoke and 82% in Bagusa earned their living from fishing. More than half of the sampled households in both villages, 57% in Yoke and 65% in Bagusa, collected NTFPs such as sago, areca palm and vegetables from the forests. On the other hand, relatively fewer households were involved in hunting and gathering (25% in Yoke and 12% in Bagusa) and selling forest products such as areca palm nut, *papeda* (a sago based porridge), and salted dried fish (36% in Yoke and 29% in Bagusa).

In all three villages in West Kalimantan, the major forest related livelihoods were harvesting timber and NTFPs, shifting cultivation and artisanal gold mining. Harvesting timber was only dominant in Nanga Jemah (60% of households), which has the largest forest area compared to the other two villages. Between 81–99% of households (in all three villages) collected NTFPs for their livelihoods. The main NTFPs were rubber followed, to a lesser extent, by seasonal *tengkawang* nuts and durian. Shifting cultivation was another livelihood activity conducted mainly for subsistence; this activity was carried out by 36–58% of households in all three villages. The percentages of households involved in artisanal gold mining varied across villages with 39% in Hulu Pengkadan, 43% in Nanga Jemah and 75% in Sri Wangi. The other forest dependent livelihoods carried out by fewer households were hunting and gathering, fishing, farming, animal husbandry, fish farming and working as wage labor in forest related activities.

In both villages in Central Java, the main forest related livelihoods were more diverse including harvesting timber from their own agroforests and Perhutani’s plantation forests (subject to Perhutani regulations), harvesting NTFPs, farming, animal husbandry, fish farming, trading, small enterprises working with forest products and labor in forest related activities. Between 41–52% of households in Lebak and Karanganyar harvested timber from Perhutani plantation forests and privately owned agroforests. Around half of the households in both villages (51% and 57%) collected NTFPs such as pine resin from Perhutani's forest (part of a profit sharing scheme between Perhutani and the local community), rudraksha jenitri seeds and *duku* (*Lansium parasiticum*) for their livelihoods. Farming was dominant in Lebak (78% of households) compared to Karanganyar (44% of households). While between 41–42% of households derived their livelihoods from animal husbandry and fish farming in both villages, relatively fewer households were involved in trading palm sugar and timber (24% in Lebak and 10% in Karanganyar) and as labor for cutting trees, carrying logs and working on farms (21% in Lebak and 28% in Karanganyar).

#### Non-forest related livelihoods

Households also relied on other livelihoods not related to the forest. These livelihoods included working outside the forest or agricultural sectors (e.g. as teachers, civil servants, traders), in-kind or cash assistance (from the government, NGOs, neighbors or relatives) and remittance (from relatives mostly working in the cities) (Fig 2). Across all the villages, noticeable proportions of households worked outside forestry and agriculture. Approximately 44–56% of households in Papua and Central Java and 29–31% in West Kalimantan worked as construction laborers, teachers, civil servants, traders or drivers. In terms of in-kind or cash assistance, between 74–79% of households in both villages in Central Java, 52–82% in both villages in Papua and 4–19% in all three villages in West Kalimantan received support from the government and, to a lesser extent, from relatives.

Migrant workers often send money to their family members in the villages. More households in Central Java (22% in Lebak and 13% in Karanganyar) received money transfers (remittance) from family members than those in West Kalimantan (2% in Nanga Jemah, 4% in Sri Wangi and none in Hulu Pengkadan).

### Local perceptions of drivers of deforestation and forest degradation

**Fig 3:**
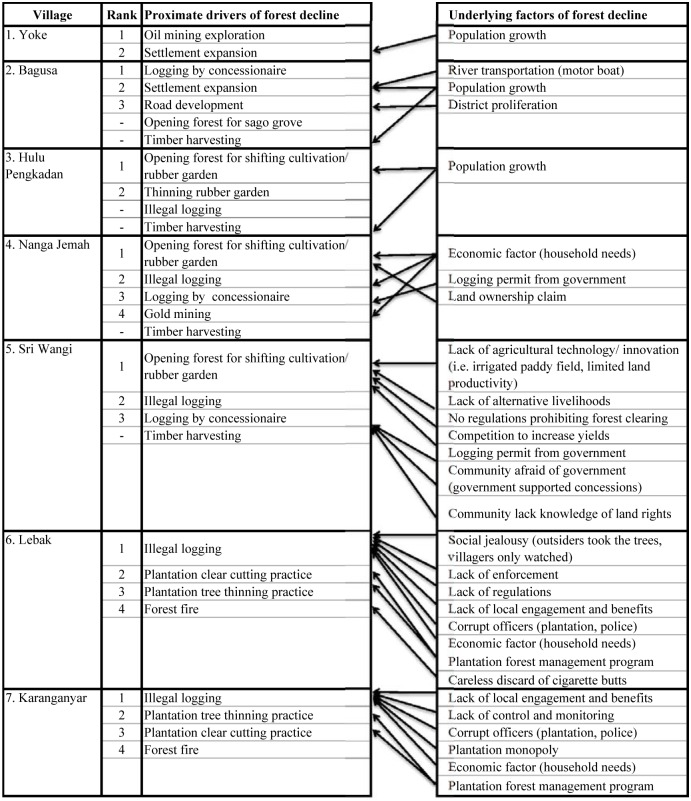
List of proximate drivers and underlying factors of forest decline in each village as described by focus group discussion participants (n = 7 FGDs). The diagrams from the FGD in each village have been amalgamated in this figure. The lists were rearranged in order of rank based on scores given by the FGD participants (the first rank is the driver with the highest score, and so on). The links between the underlying factors and the proximate drivers, as indicated by the arrows, were derived during the FGDs. Some proximate drivers are un-ranked because the participants gave no score to drivers they did not consider to have a significant impact on forest decline in comparison with the other drivers.

Village participants identified proximate drivers and to some extent the underlying factors behind the proximate drivers of deforestation and forest degradation in their villages (Fig 3).

#### Drivers in Papua

Oil exploration and logging by concessionaries ranked first as a major cause of forest decline in Yoke and Bagusa respectively (Fig 3). In the past (1980’s) Shell Indonesia Mamberamo oil company cut down trees to make way for roads, camp and oil exploration. Even though the forest has since regrown, the participants mentioned that the forest has not returned to its former condition. Recently, the participants have observed private investors’ increasing interest in exploring the area for natural gas and coal. Mamberamo Alas Mandiri (MAM) logging concessionaire has been cutting trees from the natural forest around the villages since 2000. The logging activities were perceived as a major cause of forest decline in the area, particularly in Bagusa. The concessionaire holds a license to log an area of over 670,000 hectares in Papua, the largest single logging concession in Indonesia [38].

Settlement expansion was ranked as the second cause of forest decline in both villages (Fig 3). In Bagusa road development and clearing forest for sago groves were other causes mentioned. These drivers were related to population growth, an underlying factor mentioned in both villages. Another underlying factor is river transportation, which was mentioned only in Bagusa, because it provides better access for people to come and go from the village, which is located in the Mamberamo River watershed.

#### Drivers in West Kalimantan

Clearing forest for shifting cultivation and rubber gardens ranked as the top DD drivers in all three villages in West Kalimantan (Fig 3). Shifting cultivation and rubber gardens are practiced within a rotation of agricultural land use; people open forest or fallow and burn the land for dried rice cultivation. Subsequently, after about 3 years of cultivation, the soil loses its fertility and is either planted with rubber or left fallow for at least 4 years before it can be used again or for a longer period until it re-grows into forest. Once the rubber is no longer productive (approximately 30 years) the trees are cut down and the land is burnt for another rotation.

Illegal logging and logging concessions were considered the second and third causes of forest decline in Nanga Jemah and Sri Wangi. The villages perceived the logging concession as one of the drivers due to the vast forest area impacted by the logging and, despite there being no longer active logging (logging operated during 1980’s) in the forest around Sri Wangi and Nanga Jemah, the concessionaire still holds a valid logging license.

Hulu Pengkadan mentioned illegal logging as one of the proximate drivers, but considered it an insignificant cause of forest decline. Most of the available trees are difficult to access, and the village regulations only allow villagers to cut down trees for personal use and prohibit outsiders from harvesting trees in the village. Interestingly, Hulu Pengkadan, which has the longest history of mixed rubber agroforestry practices compared to the other two villages, counted the thinning of rubber agroforests as the second cause of forest decline.

Timber harvesting was mentioned as one of the drivers of forest decline in all three villages, yet it was considered insignificant in terms of impact on forest cover because villagers only harvest a limited number of trees for housing and bridge materials. Gold mining was described in Nanga Jemah as a minor cause because it was artisanal and only involved clearing a small area along the river where the mining was taking place.

The underlying factors effecting DD varied across all study villages; population growth was identified as an underlying cause in Hulu Pengkadan whereas the local economy (households’ need for cash income), policy (concession license issuance) and land claim practices (the land is owned by those who first cleared the forest) were underlying factors in Nanga Jemah. In Sri Wangi, the underlying causes were policy and institutional factors (concession license issuance and lack of regulations), social (community afraid of the government, lack of understanding of community rights, competition among farmers), economic factors (lack of alternative livelihoods), and absence of new agricultural technologies (irrigated paddy fields and limited land productivity).

#### Drivers in Central Java

Both villages in Central Java mentioned illegal logging as the main driver of forest decline (Fig 3). Illegal logging refers to the massive logging event during the economic crisis and the change in the Indonesian government from a centralistic to decentralized system in 1998–2003. Since then, the local communities have been more involved in taking care of the Perhutani forest plantations in return for land use rights for intercropping and profit sharing.

Interestingly, the participants identified the forest plantation management practices of clear cutting and thinning trees as the drivers of forest decline and ranked them as the second and third most important drivers, respectively. Forest fire was also mentioned and placed as the least important driver. Their last major fire occurred well before the 1980’s and both natural events and human activities were mentioned as possible causes of the fire.

The underlying causes included policy and institutional factors (i.e. lack of regulations, enforcement, control and monitoring, corrupt officers, and lack of local engagement in forest plantation management) and economy factors (households’ need for cash income). In Lebak, participants mentioned local social jealousy of outsiders who illegally logged trees in the Perhutani forest area around their village as an underlying factor driving the local people to participate in illegal logging.

### Relative importance of drivers of deforestation and forest degradation for local livelihoods

Some drivers of DD were related to livelihoods and played important roles in supporting the local households. The relative importance of these livelihoods can be seen from how many households in a village rely on each livelihood and its contribution to all of the households’ livelihoods (main or secondary livelihoods).

#### Links between drivers and local livelihoods

There were four types of local livelihood activities related to drivers of DD, identified in the villages, i.e. establishment of rubber garden or sago grove, shifting cultivation, timber extraction and mining (Table 1). These four activities are related to drivers of DD in that the activities often include the clearing of a forested area or cutting of trees from either natural forests or forest plantations.

**Table 1:** List of livelihoods that are also drivers of deforestation and forest degradation.

Related local livelihoods	Driver clusters
Establishment of rubber garden or sago grove	**Proximate**: rubber garden or sago grove, thinning rubber garden.
	**Underlying:** population growth, economic factors, land ownership claim.
Shifting cultivation	**Proximate:** shifting cultivation/garden.
	**Underlying:** population growth, economic factors, land ownership claim, lack of new agricultural technology, lack of alternative livelihoods, no regulation, competition to increase yields, limited dried rice productivity.
Timber extraction	**Proximate:** logging concession, illegal logging, settlement expansion, road development, plantation clear cutting, and practice of thinning plantation trees.
	**Underlying:** population growth, river transportation, economic factors, no regulation, logging permit, community afraid of government, community do not know their land rights, lack of alternative livelihoods, no regulation, plantation program, social jealousy, lack of local engagement, corrupt officials, lack of control and monitoring, Perhutani monopoly.
Mining	**Proximate:** artisanal gold mining, oil exploration.
	**Underlying:** economic factors, no regulation.

#### Relative importance of drivers for household and village livelihoods

**Fig 4:**
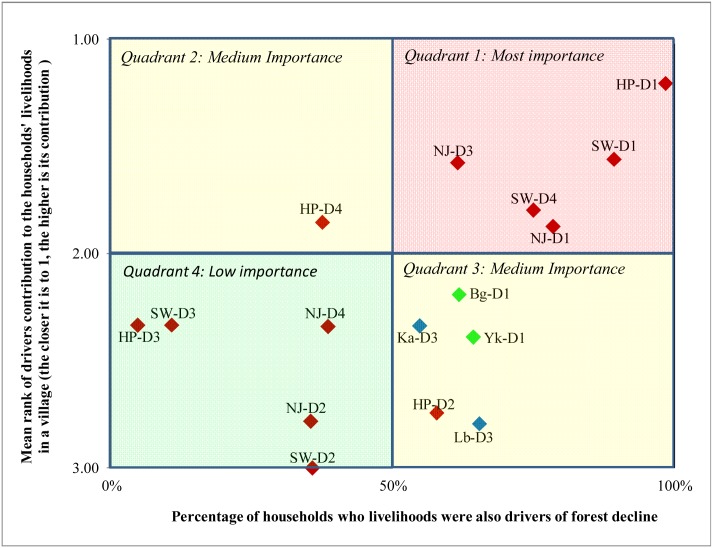
Relative importance of drivers of deforestation and forest degradation for household and village livelihoods. Villages: Yk: Yoke, Bg: Bagusa, HP: Hulu Pengkadan, SW: Sri Wangi, NJ: Nanga Jemah, Lb: Lebak, Ka: Karanganyar. Drivers: D1: Rubber or sago garden, D2: Shifting cultivation, D3: Timber extraction, D4: Mining.

Relative importance of DD drivers for household and village livelihoods is defined by: 1) the proportion of households in a village, the livelihoods of which are also drivers of DD; and 2) the mean rank of a livelihood (driver) in the households’ livelihoods in a village. Fig 4 presents the relative importance of each livelihood (driver) for household and village livelihoods in four quadrants.

The area in the top right (red shaded quadrant 1) shows drivers with the highest relative importance for household and village livelihoods. Drivers are the livelihood activities for the majority of households in the villages and the households’ main source of livelihoods. These drivers include the establishment of rubber garden (D1) in Hulu Pengkadan, Sri Wangi, and Nanga Jemah, timber extraction (D3) in Nanga Jemah, and gold mining (D4) in Sri Wangi.

The area in the top left and bottom right (yellow shaded quadrants 2 and 3) describe drivers with medium relative importance for household and village livelihoods. Drivers in the bottom right are carried out by more than half of the households in the village, but serve as second or third sources of household livelihoods, while drivers in the top left are primary sources of livelihoods carried out by less than half the households in the village. Drivers that are included in these areas are sago grove (D1) in Yoke and Bagusa, shifting cultivation (D2) in Hulu Pengkadan, timber extraction (D3) in Lebak and Karanganyar and gold mining (D4) in Hulu Pengkadan.

Moreover, the area in the bottom left (green shaded quadrant 4) consists of drivers with relatively lower importance for the household and village livelihoods. Drivers are associated with the livelihoods of less than half the households in the village and are the second or third sources of household livelihoods. This area consists of shifting cultivation (D2) in Sri Wangi and Nanga Jemah, timber extraction (D3) in Hulu Pengkadan and Sri Wangi, and gold mining (D4) in Nanga Jemah.

## Discussion: Opportunities for Local Participation in REDD+ and MRV

### Local perceptions for better understanding of drivers

Most studies on drivers of DD occur at global, regional or national levels. While they provide an interesting overview of drivers, the level of these studies is broad and not comprehensive enough to use as a basis for policy intervention. For instance, Kissinger et al. [19] highlight commercial drivers, agriculture and wood extraction, as the main DD drivers in Southeast Asia together with cross-scale underlying factors of international markets and prices, national policies, domestic markets, governance, population growth, and local poverty and subsistence. Hosonuma et al. [16] limited their assessment to proximate drivers, and presents major drivers of deforestation (i.e. commercial and subsistence agriculture, mining, infrastructure, and urban expansion) and degradation (i.e. logging, uncontrolled fires, fuel wood collection, charcoal production, and livestock grazing) in developing countries, including Indonesia. Another analysis that utilized local case studies to build a picture of DD at the regional level, found agriculture expansion, infrastructure extension and wood extraction to be the key proximate causes of deforestation, driven by a set of economic, political, and institutional factors [17]. At the national level, Indrarto et al [20] identify country specific drivers in Indonesia that include oil palm plantations, mining, illegal logging, forest fires and swidden agriculture (proximate drivers) and resource-based economic development, timber market, population growth, tenure, local politics, poor governance and forest management (underlying factors).

Studies of drivers at the sub-national level are few. Curran et al. [21] specifically gathered information on drivers at the village level and contrasts them with a literature review of drivers elsewhere. This provides place-specific proximate drivers (i.e. illegal logging, issuance of small parcel leases within protected areas, and conversion to oil palm) that link to specific underlying drivers (i.e. international markets, oil palm plantation expansion, government policies, and international finance institutes). However, this analysis is currently limited to drivers within protected areas in Kalimantan.

This study reveals that local processes behind forest cover changes are dynamic and varied from place to place. They are driven by various cross-scale proximate drivers and underlying factors (Fig 4). The main drivers in Papua came from outside of the villages (i.e. mining exploration and logging concessions). In Java, illegal logging, conducted by both villagers and outsiders in Perhutani forest plantations, was considered the main driver. In West Kalimantan, DD were related to agricultural activities (i.e. shifting cultivation and smallholder rubber plantations). Population growth was perceived as the underlying factor in both villages in Papua, while in West Kalimantan and Java the underlying causes were a mix of population growth, policies and institutional factors, technological interventions in forestry and agricultural sectors, and economic factors.

Drivers in this study were identified based on local perceptions of the drivers of DD processes taking place in their village areas over a 30 year time period. Many of these drivers are still happening today such as illegal logging, shifting cultivation, rubber plantations, and timber extraction. Some are past events such as the vast illegal logging in Central Java, oil exploration in Papua, and logging concessions in West Kalimantan. Even though these were drivers in the past, to some extent, the local people (at the time of our study) were still affected by the loss of the forest; and they perceived possible risks from similar types of drivers in the future. In the villages in West Kalimantan, the concessionaire still holds the legal right to log the forest; in Papua, the community observed the increasing interest of investors to explore the area for coal and natural gas; in Central Java, the communities felt uncertain about the continuity of the partnership between the communities and state-owned forest plantation that has so far prevented outsiders from illegally logging the forest.

The place-specific nature of drivers implies that measures to address DD should be developed case by case and based on a detailed understanding of drivers in each respective location. The local communities can provide in-depth insights into drivers and the dynamic DD processes at the local level such as:

1. The different ways livelihood activities act as drivers (actors, proximate drivers and underlying factors, historical and current DD): this could be used to see who will be affected and how, who to target, scale of drivers, and possible future risks of DD.
2. The importance of these drivers for local livelihoods (prevalence and level of importance of a driver): this could be used to assess the risks associated with addressing drivers, opportunity costs, possible benefits and co-benefits, and safeguards.

### Considering local livelihoods in efforts to reduce deforestation and forest degradation

Local communities, to varying degrees, rely on forest for their livelihoods (Fig 2). Some of their livelihood activities involve modification and conversion of land and forest, many of which are considered drivers of deforestation and forest degradation. The local drivers’ contribution to national deforestation and forest degradation is considered low in Indonesia compared to other countries (local drivers contributed less than 33% to national DD) [5], yet it is continuous and widespread across forest areas.

In the context of locally driven DD, addressing the drivers and actors behind them might be the most important and easiest measure to take for REDD+ [39]. This approach requires local participation to change their forest and land uses that cause DD. However, local people utilize the forest mainly to fulfill their livelihood needs, thus solely prioritizing drivers based on the scale of forest cover change and carbon emissions is not enough. REDD+ must take into consideration the relative importance of drivers for local livelihoods. For instance, addressing rubber plantations in West Kalimantan might be seen as a priority due to the high impact the plantation has on land cover change. However, it might not be the most efficient and effective approach in terms of risks and opportunity costs. Communities are unlikely to participate in activities that prevent them from carrying out their livelihood activities. Thus, interventions should include incentive(s) to compensate affected communities for a lost livelihood [4, 40, 41]. In addition, participation in MRV activities can also serve as alternative job opportunities for those whose livelihoods are affected by the interventions [1, 42]. Both compensation and provision of jobs should be undertaken with an understanding of the role of the livelihood in each village.

We found that livelihoods acted as drivers differently in each place (Fig 4). Two measures of this difference are the percentage of the population involved in a livelihood activity and the ranking of the income that comes from that activity (primary, secondary, or third source). For example, timber extraction (Java) and sago cultivation (Papua) are carried out by more than half of the villagers, but they serve as secondary or third sources of livelihoods. In West Kalimantan villages, the drivers are the livelihoods of more than half of the village and act as a primary source of income (i.e. rubber gardens in all the villages, timber extraction in Nanga Jemah, and artisanal gold mining in Sri Wangi). The place-specific nature of drivers and their links to local livelihoods implies that no one measure fits all for addressing and monitoring drivers of DD. In the context of participatory MRV, it shows one type of information local communities can provide. This information is required to prioritize areas for interventions and monitoring of both drivers and livelihood impacts of interventions, as well as for adaptive management of an intervention. Detailed information on drivers and their links to local livelihoods is also useful to shape the incentive structure, and ensure clear, fair and effective distribution of incentives. Incentives for participation in MRV can be fashioned in accordance with how and whose local livelihoods are affected by REDD+ measures. The more important an affected livelihood is for the local communities the more incentive(s) should be built into the activities. As alternative jobs, participatory MRV activities should target the households whose livelihoods are most affected. Compensating communities for the monitoring work [12], ensuring a clear incentive structure [43], and equitable distribution of incentives [44] are key factors for higher and more sustainable local participation in MRV. When livelihoods are considered in the participatory MRV activities, local participation has three functions: 1) ensures effectiveness [45], transparency and accountability [46] of REDD+ activities on the ground; 2) provides a way to distribute benefits (livelihood opportunities) [47, 48]; and 3) complies with social safeguards.

## Conclusion

Factors that influence forest cover change vary from place to place, come from multiple sectors (e.g. forest, agriculture, mining and infrastructure), and have cross-level aspects (local, sub-national and national). Thus, local level analysis of drivers is necessary to better capture these varieties and to understand the dynamics of local land use activities. This analysis also needs to be embedded into land use studies more generally so that the focus is not only on the forestry sector. This locally collected data could inform, supplement and validate other sources of data (e.g. remote sensing and forestry) and should be integrated into the national level analysis to provide comprehensive assessment of drivers.

We argue that understanding drivers of DD from the local communities’ perspectives is essential in the design of interventions and monitoring systems that are locally appropriate. In the early phase of intervention development, prioritizing drivers to address DD should not be based only on the scale of forest cover change but also on the relative importance of drivers for local livelihoods.

We presented how locally collected data can shed light on the different ways livelihoods act as drivers and the relative importance of these drivers for the local people. This information can be used to assess the risks, opportunity costs, possible benefits, actors and beneficiaries associated with activities to reduce deforestation and forest degradation. It also highlights possible areas for local participation, i.e. providing information on the locally driven activities that led to DD and monitoring the drivers and local DD processes. From the project development perspective, local participation also serves as a mechanism to ensure compliance with social safeguards. Incorporating locally collected data on drivers in the design and monitoring of interventions will be crucial in ensuring outcomes that benefit not only forests but also local people.

## References

[1] Palmer Fry B. Community forest monitoring in REDD+: the ‘M’in MRV? Environ Sci Policy. 2011;14(2):181–7.

[2] Brewster J, Bradley A, Yeang D. Community-based Monitoring, Reporting and Verification (MRV): An assessment in the Oddar Meanchey Community Forestry REDD+ Site, Cambodia. 2012.

[3] Skutsch MM, van Laake PE, Zahabu EM, Karky BS, Phartiyal P. Community monitoring in REDD. Realising REDD. 2009:101.

[4] Danielsen F, Skutsch M, Burgess ND, Jensen PM, Andrianandrasana H, Karky B, et al At the heart of REDD+: a role for local people in monitoring forests? Conservation Letters. 2011;4(2):158–67.

[5] Pratihast AK, Herold M, De Sy V, Murdiyarso D, Skutsch M. Linking community-based and national REDD plus monitoring: a review of the potential. Carbon Manag. 2013;4(1):91–104.

[6] Peters-Guarin G, McCall MK. Community Carbon Forestry (CCF) for REDD: Using CyberTracker for Mapping and Visualising of Community Forest Management in the Context of REDD. 2010.

[7] Rana EB, Shrestha HL, Silwal R. Participatory carbon estimation in community forest: methodologies and learnings. The Initiation. 2008;2(1):91–8.

[8] McCall K. Local participation in mapping, measuring and monitoring for community carbon forestry. Community Forest Monitoring for the Carbon Market: Opportunities Under REDD. 2010.

[9] Verplanke J, Zahabu E. A field guide for assessing and monitoring reduced forest degradation and carbon sequestration by local communities. Project team KYOTO: Think Global, Act Local, Enschede, Países Bajos 93 p 2009.

[10] Mukama K, Mustalahti I, Zahabu E. Participatory Forest Carbon Assessment and REDD: Learning from Tanzania. International Journal of Forestry Research. 2011;2012.

[11] Skutsch MM, McCall MK, Karky B, Peters-Guarin G. Case Studies on Measuring and Assessing Forest Degradation; Community Measurement of Carbon Stock Change for REDD. Rome: FAO, 2009.

[12] Larrazábal A, McCall MK, Mwampamba TH, Skutsch M. The role of community carbon monitoring for REDD+: A review of experiences. Curr Opin Env Sust. 2012;4(6):707–16.

[13] Fraser ED, Dougill AJ, Mabee WE, Reed M, McAlpine P. Bottom up and top down: Analysis of participatory processes for sustainability indicator identification as a pathway to community empowerment and sustainable environmental management. Journal of environmental management. 2006;78(2):114–27. 10.1016/j.jenvman.2005.04.009 16095806

[14] Skutsch MM. Community forest monitoring for the carbon market: opportunities under REDD: Routledge; 2011.

[15] Skutsch MM. Reducing carbon transaction costs in community-based forest management. Clim Policy. 2005;5(4):433–43.

[16] Hosonuma N, Herold M, De Sy V, De Fries RS, Brockhaus M, Verchot L, et al An assessment of deforestation and forest degradation drivers in developing countries. Environ Res Lett. 2012;7(4).

[17] Geist HJ, Lambin EF. Proximate causes and underlying driving forces of tropical deforestation. Bioscience. 2002;52(2):143–50.

[18] Houghton RA. Carbon emissions and the drivers of deforestation and forest degradation in the tropics. Curr Opin Env Sust. 2012;4(6):597–603.

[19] Kissinger G, Herold M, De Sy V. Drivers of Deforestation and Forest Degradation: A Synthesis Report for REDD+ Policymakers. 2012.

[20] Indrarto GB, Murharjanti P, Khatarina J, Pulungan I, Ivalerina F, Rahman J, et al The context of REDD+ in Indonesia. Bogor, Indonesia: Center for International Forestry Research (CIFOR); 2012.

[21] Curran LM, Trigg SN, McDonald AK, Astiani D, Hardiono YM, Siregar P, et al Lowland forest loss in protected areas of Indonesian Borneo. Science. 2004;303(5660):1000–3. 10.1126/science.1091714 14963327

[22] Indrabudi H, De Gier A, Fresco LO. Deforestation and its driving forces: A case study of Riam Kanan watershed, Indonesia. Land Degrad Dev. 1998;9(4):311–22.

[23] Langner A, Miettinen J, Siegert F. Land cover change 2002–2005 in Borneo and the role of fire derived from MODIS imagery. Global Change Biol. 2007;13(11):2329–40.

[24] Romijn E, Ainembabazi JH, Wijaya A, Herold M, Angelsen A, Verchot L, et al Exploring different forest definitions and their impact on developing REDD plus reference emission levels: A case study for Indonesia. Environ Sci Policy. 2013;33:246–59.

[25] Gibbs HK, Brown S, Niles JO, Foley JA. Monitoring and estimating tropical forest carbon stocks: making REDD a reality. Environ Res Lett. 2007;2(4).

[26] Sanchez-Azofeifa GA, Castro-Esau KL, Kurz WA, Joyce A. Monitoring carbon stocks in the tropics and the remote sensing operational limitations: from local to regional projects. Ecol Appl. 2009;19(2):480–94. 19323204 10.1890/08-1149.1

[27] Olander LP, Gibbs HK, Steininger M, Swenson JJ, Murray BC. Reference scenarios for deforestation and forest degradation in support of REDD: a review of data and methods. Environ Res Lett. 2008;3(2):025011.

[28] Peres CA, Barlow J, Laurance WF. Detecting anthropogenic disturbance in tropical forests. Trends in Ecology & Evolution. 2006;21(5):227–9. 16697907 10.1016/j.tree.2006.03.007

[29] REDD+ Task Force MRV Working Group. Strategy and Implementation Plan for REDD+ Measurement, Monitoring, Reporting, and Verification (MRV) in Indonesia. In: Force RT, editor. v.1_11 November 2012 ed Jakarta: Satuan Tugas Persiapan Kelembagaan REDD+; 2012 p. xv + 106.

[30] Salvini G, Herold M, De Sy V, Kissinger G, Brockhaus M, Skutsch M. How countries link REDD+ interventions to drivers in their readiness plans: Implications for monitoring systems. Environ Res Lett. 2014;9(7):074004.

[31] Joseph S, Herold M, Sunderlin WD, Verchot LV. REDD plus readiness: early insights on monitoring, reporting and verification systems of project developers. Environ Res Lett. 2013;8(3).

[32] Boissière M, Beaudoin G, Hofstee C, Rafanoharana S. Participating in REDD+ Measurement, Reporting, and Verification (PMRV): Opportunities for Local People? Forests. 2014;5(8):1855–78.

[33] Kothari CR. Research methodology: methods and techniques: New Age International; 2011.

[34] Microsoft. Microsoft Excel. 2010.

[35] Byron N, Arnold M. What futures for the people of the tropical forests? World development. 1999;27(5):789–805.

[36] Fisher RJ, Srimongkontip S, Veer C. People and forests in Asia and the Pacific: situation and prospects Asia-Pacific Forestry Towards 2010. Asia-Pacific Forestry Sector Outlook Study Working Paper Series (FAO) 1997.

[37] Huang R. RQDA: R-based Qualitative Data Analysis. R package version 2.15.3. URL< http://rqda.r; 2013.

[38] Murdiyarso D, Kurnianto S. Ecohydrology of the Mamberamo basin: an initial assessment of biophysical processes. 2008.

[39] Skutsch M, Torres AB, Mwampamba TH, Ghilardi A, Herold M. Dealing with locally-driven degradation: A quick start option under REDD+. Carbon balance and management. 2011;6:16 10.1186/1750-0680-6-16 22204698 PMC3339330

[40] Chhatre A, Agrawal A. Trade-offs and synergies between carbon storage and livelihood benefits from forest commons. Proceedings of the National Academy of Sciences. 2009;106(42):17667–70. 10.1073/pnas.0905308106 PMC2764886 19815522

[41] Blom B, Sunderland T, Murdiyarso D. Getting REDD to work locally: lessons learned from integrated conservation and development projects. Environ Sci Policy. 2010;13(2):164–72.

[42] Hawthorne SD, Boissière M. Literature review of participatory measurement, reporting and verification (PMRV): CIFOR; 2014. 10.1371/journal.pone.0157826 PMC5094709 27812110

[43] Holck MH. Participatory forest monitoring: an assessment of the accuracy of simple cost—effective methods. Biodivers Conserv. 2008;17(8):2023–36.

[44] Christie P, Lowry K, White AT, Oracion EG, Sievanen L, Pomeroy RS, et al Key findings from a multidisciplinary examination of integrated coastal management process sustainability. Ocean & Coastal Management. 2005;48(3):468–83.

[45] Lederer M. From CDM to REDD+—What do we know for setting up effective and legitimate carbon governance? Ecol Econ. 2011;70(11):1900–7.

[46] Lawlor K, Weinthal E, Olander L. Institutions and policies to protect rural livelihoods in REDD+ regimes. Global Environmental Politics. 2010;10(4):1–11.

[47] Chhatre A, Lakhanpal S, Larson AM, Nelson F, Ojha H, Rao J. Social safeguards and co-benefits in REDD+: a review of the adjacent possible. Curr Opin Env Sust. 2012;4(6):654–60.

[48] Visseren-Hamakers IJ, McDermott C, Vijge MJ, Cashore B. Trade-offs, co-benefits and safeguards: current debates on the breadth of REDD+. Curr Opin Env Sust. 2012;4(6):646–53.

